# Topping and NPK fertilization alter seed germination, plant growth and active components of *Valeriana amurensis*

**DOI:** 10.3389/fpls.2024.1430507

**Published:** 2024-07-03

**Authors:** Junkai Wu, Dayong Leng, Jinhai Huo, Ruoquan Zhang, Xiaowei Du

**Affiliations:** ^1^ School of Pharmacy, Quanzhou Medical College, Quanzhou, China; ^2^ Key Laboratory of Chinese Materia Medica, Ministry of Education, Pharmaceutical College, Heilongjiang University of Chinese Medicine, Harbin, China; ^3^ Institute of Chinese Materia Medica, Heilongjiang Academy of Chinese Medicine Sciences, Harbin, China; ^4^ TCM Department, Zhenbaodao Pharmaceutical Co. Ltd., Hulin, China

**Keywords:** *Valeriana amurensis* Smir. ex Kom., seed germination, pinching, valtrate, valepotriates, essential oil

## Abstract

**Introduction:**

*Valeriana amurensis*, a tall herbaceous plant, has been traditionally utilized as a herbal remedy both in China and Russia.

**Methods:**

This study was set to explore how to cultivate high quality seedlings, considering factors such as seedling time, seeding density, shading, and plastic film mulching. In addition, we investigated the impact of topping and fertilizer on the growth and quality of *V. amurensis*.

**Results:**

According to the percentage of seed germination, the seeds of *V. amurensis* were sowed with 0.88 g m^-2^ density using plastic film mulching from late April to mid-May (germination percentage was more than 30%). The optimal Transplanting time was identified as late September, ensuring a high survival rate of 98%. Generally, topping showed the most improved growth indices in late fruit period (21.87 for number of radical leaves) and withering period (6.50 for number of buds and 234.81 for number of roots). Topping increased the yields of valtrate (10.91 mg per plant), valepotriates (809.51 mg per plant) and essential oil (395.64 mg per plant) in withering period. Nitrogen fertilizer promoted maximum root growth and increased the biomass of *V. amurensis*. Meanwhile, N fertilizer significantly increased the yields of valtrate to 10.46 mg per plant and valepotriates to 772.32 mg per plant among three types of fertilizers. Seedlings are obtained according to rational sowing factors and transplanting time. Topping and nitrogen fertilization emerge as superior strategies to enhance the growth and medicinal quality of this valuable plant.

**Discussion:**

This study provides actionable insights for the cultivation *V. amurensis*.

## 1 Introduction

The genus *Valeriana* originates from North America, Asia, and Europe and contains about 315 species distributed throughout the world (Das et al., 2021). Many compounds including sesquiterpene, monoterpene, valepotriate, lignan, alkaloid, etc, have been isolated and identified during the last 130 years (Wu et al., 2014; Erdoğan et al., 2022; Quan et al., 2022). The active components possess sedative, antidepressant, antianxiety, anticonvulsant, and antimicrobial properties (Jugran et al., 2019; Sen-Utsukarci et al., 2019; Wang et al., 2020).

*Valeriana amurensis* Smir. ex Kom., commonly known as “valerian” in the Northeast of China, has been used to treat neurological system diseases for thousands of years in Chinese medicine (Zhu, 1989). However, increasing demand for natural tranquilizer has led to a rapid depletion of *V. amurensis*. Therefore, cultivation is a feasible approach to maintain resource of *V. amurensis*, while also allowing greater quality control of the medicinal plant. However, the seeds of *V. amurensis* possess dormant characteristics and low percentage of germination in the wild condition. In our previous study, the influences of concentration and duration of gibberellin treatment, and time of cold stratification on seed germination rates were investigated using orthogonal tests (Zhang, 2010). The maximum seed germination was recorded at 78% with 20-day cold stratification after 600 mg L^-1^ gibberellin for 24 hours.

Plastic film mulch plays an important role in promoting plant growth. Yields of strawberries, potato and Chinese cabbage could be increased significantly by plastic film mulch dependent on different environmental conditions (Li et al., 2021; Liu et al., 2022; Morra et al., 2022). Plastic film mulch is particularly effective practice for improving soil water retention, and increasing plant yield (Adhikari et al., 2016; Ma et al., 2018). A series of studies has shown that plastic film mulching promotes the water plant consumption, due to a reduction in the evaporation process in soil surface combined with improved physiological process of plant transpiration (Daryanto et al., 2017; Ding et al., 2018). In addition, plastic film mulching can elevate surface temperature, promote soil nutrient turnover, alter the distribution of nitrate in the soil profile, and promote the uptake of nitrogen (N) by roots (Yang et al., 2018; Jiang et al., 2018b). However, plastic film mulching might cause too higher temperature, whereas its soil shading effects can avoid seedling burned and maintains suitable soil environment for long term. Therefore, plastic film mulch combined with shading promotes plant growth and increases plant yield.

Fertilization is one of the standard treatments to favor early plant growth, N, phosphorus (P), and potassium (K) are essential nutrients for plants. Nitrogen plays an important role in plant cell division during growth periods, and optimal N levels can significantly enhance the growth of plants such as sorghum, soybean, maize and safflower (Hao et al., 2014; La Menza et al., 2017; Mohammad and Ali, 2018; Srivastava et al., 2018; Tsuji et al., 2023). Phosphorus fertilizer promotes root growth, drought tolerance, disease resistance, and enhances nutrient and water uptake by seedlings (Kang et al., 2023). Potassium fertilizer enhances osmotic cell regulation, participates in photosynthesis, improves sugar metabolism, enhances drought resistance, and increases yield (Fang et al., 2024). However, the effects of N, P, K fertilizers on the growth of *V. amurensis* remain unclear.

The primary objective of our study was to conduct a comprehensive assessment of the factors influencing the seed germination of *V. amurensis*. Specifically, we examined the effects of seedling timing, seeding density, shading, and the use of plastic film mulching on germination efficiency. Our secondary aim was to determine the impact of transplanting time on the survival rate of these seedlings. Furthermore, we sought to identify optimal agricultural practices, particularly regarding topping techniques and fertilization strategies, with the goal of enhancing both the yield and the bioactive constituent content in the roots and rhizomes of *V. amurensis*. Our research introduces a novel approach by integrating a multi-factorial analysis to assess the complex interplay between environmental and agricultural factors on the growth and development of *V. amurensis*. The systematic evaluation of these factors, as well as the optimization of farming methods, represents an advancement in the field. The findings of our study not only contribute to the fundamental understanding of *V. amurensis* cultivation but also offer practical insights for the herbal medicine utilization.

## 2 Materials and methods

### 2.1 Experimental site

Field experiments were conducted over three successive growing seasons (2018 to 2020) at the Qinghe seed and seedling Breeding Base of Heilongjiang University of Chinese Medicine, located in Heilongjiang Province, China (46°21′19″N, 129°22′27″E, and 348 m above sea level) with a typical hill continental climate. The climate is characterized by a cold and extended winter, and a bried, refreshing summer. The soil at the experimental site had the following characteristics: a pH of 5.82, and the contents of organic matter, total N, available P and exchangeable K measured at 80.23 g kg^-1^, 2.45 g kg^-1^, 2.35 g kg^-1^ and 10.79 g kg^-1^, respectively.

### 2.2 Experimental design and practice

#### 2.2.1 Experimental design

**Figure 1:**
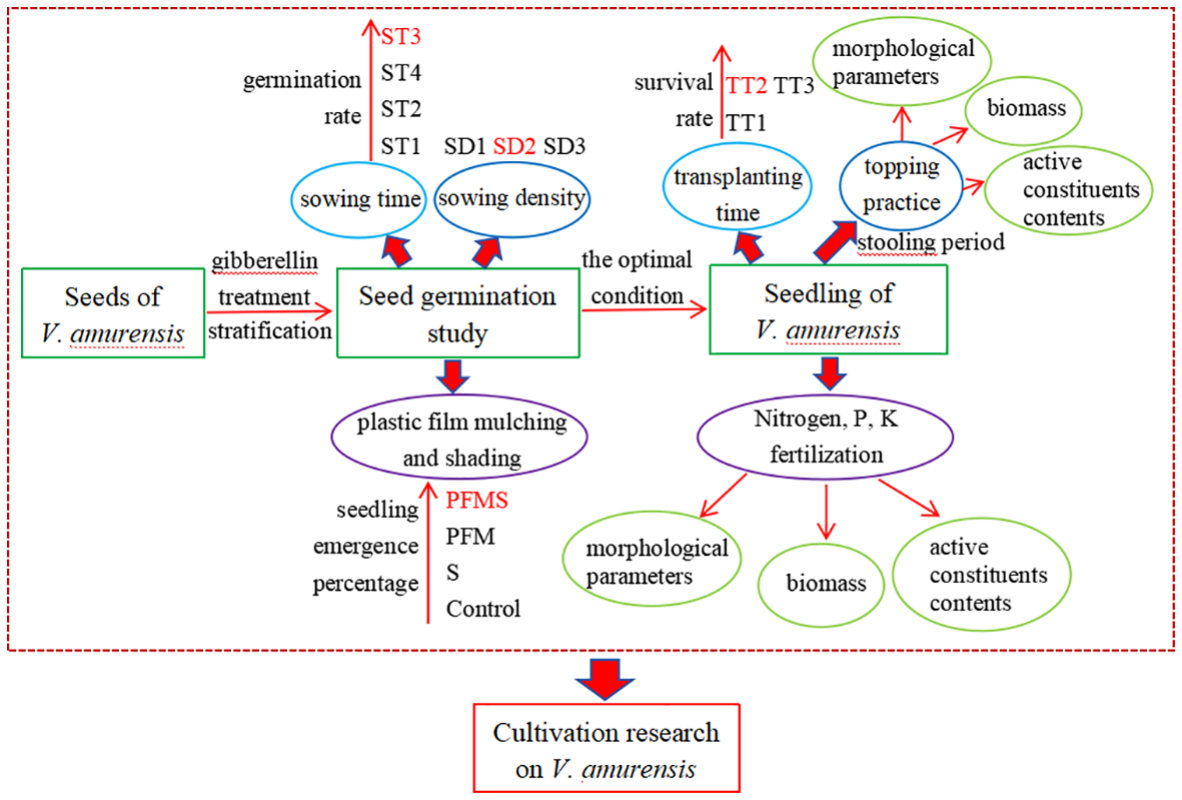
Schematic presentation of cultivation research on *V. amurensis*.

The experimental design was a randomized block layout with three replicates for each treatment (**Figure 1**). Four sowing time (ST) treatments were established: October 17, 2018 (ST1), April 7, 2019 (ST2), April 27, 2019 (ST3), and May 20, 2019 (ST4). Seedbeds of 1 m × 10 m were used to grow the seeds from October 17, 2018 to May 20, 2019. Three seeding densities (SD) were implemented: 0.44 g m^-2^ (SD1), 0.88 g m^-2^ (SD2) and 1.76 g m^-2^ (SD3). The experimental treatments included four different combinations of plastic film mulching and shading: (1) plastic film mulching (LLDPE-9085, Tianjin Petroleum Corporation Co., Ltd., China) combined with shading (SIW-16, Anping County Xingtai Net Industry Co., Ltd China) (PFMS); (2) plastic film mulching alone (PFM); (3) shading alone (S); (4) a control with neither plastic film mulching nor shading (control). These treatments were applied in 2019.

**Figure 2:**
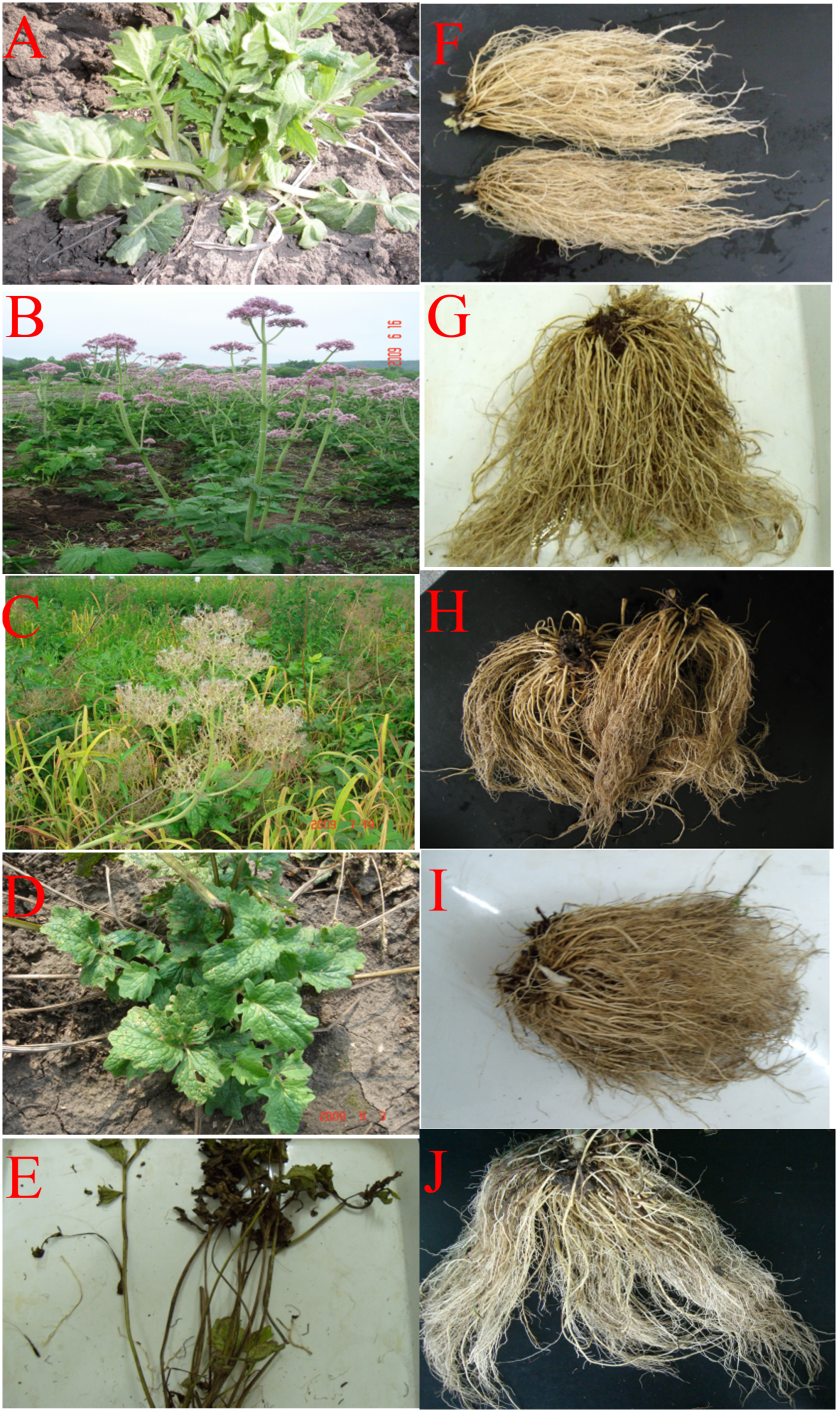
Plant morphology, roots and rhizomes of *V. amurensis* in different growth periods. **(A–E)** Show plant morphology of *V. amurensis* in stooling period, flowering period, fruit ripening period, late fruit period and withering period respectively; **(F–J)** show roots and rhizomes of *V. amurensis* in stooling period, flowering period, fruit ripening period, late fruit period and withering period respectively.

Three transplanting time (TT) were chosen: early July 2019 (TT1), lately September 2019 (TT2), and early April 2020 (TT3); note: seedlings were selected from same seedbed. Following several months of growth, seedlings of nearly uniform size were carefully selected and transplanted into treatment plots at different times from 2019 to 2020. The topping time was set as stooling period (May 17, 2019) and thirty plants were chosen from the control group at random. The little leaf was the tip of the lowest leaf lobe. The length and width of the top leaf, length and width of the little leaf, length of the petiole and the stem length were measured with tapeline respectively during both the fruit ripening period and the late fruit period. The stem and root diameters were assessed using a digital caliper at the fruit ripening period, late fruit period, and withering period (Fan, 2022). The number of radical leaves, roots and buds on *V. amurensis* plants were determined through direct observation. The fertilization treatments were based on N, P, and K fertilizer levels: 350 kg ha^-1^, 600 kg ha^-1^, 150 kg ha^-1^. The nitrogen fertilizer (total nitrogen content ≥ 50%) was provided in the form of urea and applied biannually with 300 kg ha^-1^ and 50 kg ha^-1^distributed on July 14 (stooling period) and June 17 (flowering period) respectively. Phosphorus (calcium superphosphate, P% about 16%-18%), and K (potassium sulfate, K% about 50%) fertilizers were applied in July 14 (stooling period) each year with application of 600 kg ha^-1^ and 150 kg ha^-1^. Plants were harvested at the withering period (mid-October) from 2019 to 2020, when the number of roots and buds, root length and average root diameter were measured. Plant morphology, roots and rhizomes of *V. amurensis* across various growth stages are illustrated in **Figure 2**.

#### 2.2.2 Experimental practice

Seeds of *V. amurensis* (about 3 mm in length) were collected from Qinghe County, Heilongjiang Province, China, and were identified using macroscopic identification method by Professor Chen Wang. Voucher specimens (Herbarium No. 202009–202014) had been archived in the herbarium collection of the Pharmacognosy Laboratory, College of Pharmacy, Heilongjiang University of Chinese Medicine, Harbin, Heilongjiang, China. For each replicate of the topping and the N, P, K fertilizers, 10 plants were collected for experimental measurement and analysis. The fresh biomass of the roots and rhizomes was measured. After drying the fresh roots and rhizomes to constant weight in an air dry oven (DHG-9140A, Shanghai Yiheng Technologies Co., Ltd., China) at 50°C, the dry biomass was recorded for yield calculation. For each plant, the dry biomass and the corresponding drying rate of the roots and rhizomes were determined. These dried samples were utilized for further chemical analyses.

### 2.3 Materials and reagents

A Shimadzu LC-2010A HT system with a LC-10AT Pump, a UV-Visible detector, a PDA 10A autosampler, Diamonsil C18 (5 μm, 250 mm, ID 4.6 mm, Dikma Technologies Co., Ltd., China), precolumn Hypersil C18 (5 μm, 20 mm, ID 4.6 mm) and a CLASS-VP chromatographic workstation were used for the analysis of the active pharmaceutical constituents. The mixed standard substances of valtrate and isovaltrate (1:3) were obtained from the Department of Pharmaceutical Biology, University Centre for Pharmacy, Groningen Institute for Drug Studies (GIDS), University of Groningen. HPLC-grade acetonitrile and methanol were procured from Tedia Company, and all other chemicals used were of analytical grade.

### 2.4 Essential oil extraction

The dried roots and rhizomes collected at various harvest periods were ground to a uniform particle size of 0.8 mm, through a standard mesh sieve No. 2 and after that divided into three replicates (50 g per group) at random. Essential oil was isolated via hydrodistillation according to the appendix XD of 2015 edition the *Chinese Pharmacopeia*. The oil samples were measured precisely after the removal of moisture using anhydrous sodium sulphate.

### 2.5 Study on variation of valepotriates

The contents of valepotriates were determined by potentiometric titration assay as previously described in detail (Wu et al., 2019). Standard solutions of valtrate and isovaltrate (4.3 mg mL^-1^) were made by dissolving the chemicals in methanol, then stored at -20°C until required for analysis. A sample of dried root and rhizome powder (5 g) was precisely weighed and extracted with 30 mL of chloroform for 3 times in an ultrasonic bath, with an initial extraction duration of 60 minutes followed by two additional 30-minute extractions. Then, the chloroform solutions were combined and metered volume to 100 mL with chloroform as sample solution. An aliquot (10 mL) of this solution was evaporated using a rotary evaporator (RE-52, Shanghai Broadcom Chemical Technology Co., Ltd., China). The residue was dissolved in 30 mL of acetone, then 15 mL of a 0.01 mol L^-1^ sodium hydrate solution was added, and the mixture was hydrolyzed in a water bath maintained at 56–59°C for 30 minutes. Sample solutions were titrated by 0.01 mol L^-1^ hydrochloric acid, and the end point of titration was determined according to the appendix 2015 edition *Chinese Pharmacopeia*.

### 2.6 HPLC analysis of valtrate

For the preparation of standard solutions, a mixture of valtrate and isovaltrate was dissolved in methanol to a final concentration of 0.1030 mg mL^-1^ and stored at -20°C until analysis (Wu et al., 2019). A sample of dried root and rhizome powder (1 g) was extracted with methanol solutions of 10 mL, 10 mL, 5mL separately in an ultrasonic bath for 40 min. The methanol solutions were combined and metered volume to 25 mL with methanol and filtered through a 0.45 μm filter membrane as the sample solution for HPLC analysis. A Diamonsil C18 column (5 μm, 250 mm, ID 4.6 mm, Dikma Technologies Co., Ltd., China) was employed for chromatographic separation. The UV absorption was set at 255 nm for determination of valtrate. The column temperature was controlled at 30°C. The mobile phase was a gradient elution of acetonitrile (A) and water (B), commencing with 50% A for 8 min, then rising to 60% A after 17 min, keeping isogradient 60% A to 56 min, then to 80% A after 2 min, and 90% A 2 min later. The injection volume was set as 20 μl and the flow rate was 1.0 mL min^-1^.

### 2.7 Statistical analysis

Statistical analysis of *V. amurensis* seed germination data was conducted using Statistical Analysis System (SAS) software 9.4 SAS Institute Cay N.C. (USA). And the data were analyzed by one-way ANOVA tests. The differences of groups were detected by one-way ANOVA tests. The PROC GLM procedure of SAS was employed to assess the impact of various fertilization treatments on plant morphology, as well as the effects of topping and fertilization on the yield and quality of the underground plant parts. A *P* value of less than 0.05 was considered as statistically significant.

## 3 Results

### 3.1 Effects of sowing temperature on seed germination of *V. amurensis*

**Figure 3:**
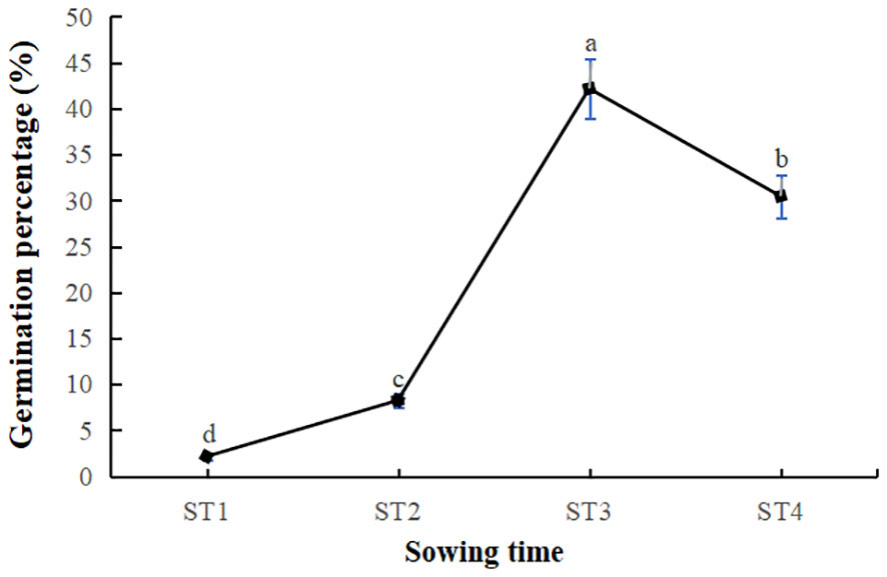
Germination percentage of *V. amurensis* on different sowing time (%). Significant differences at *p* < 0.05 are indicated by different letters.

The percentages of seed germination at different sowing times (ST1, ST2, ST3 and ST4) are presented in **Figure 3**. Germination of *V. amurensis* seeds commenced on the seventh day post-sowing for ST2, ST3 and ST4. For ST1, germination was observed only after the application of plastic film mulching and shading measures initiated on April 7, 2019. The maximum percentage of seed germination was recorded at 42.17% for ST3. In stark contrast, the ST1 treatment recorded the lowest germination rate at a mere 2.13%. These findings underscore the critical influence of sowing time on the germination success of *V. amurensis* seeds.

### 3.2 Effects of sowing density on seed germination of *V. amurensis*

**Figure 4:**
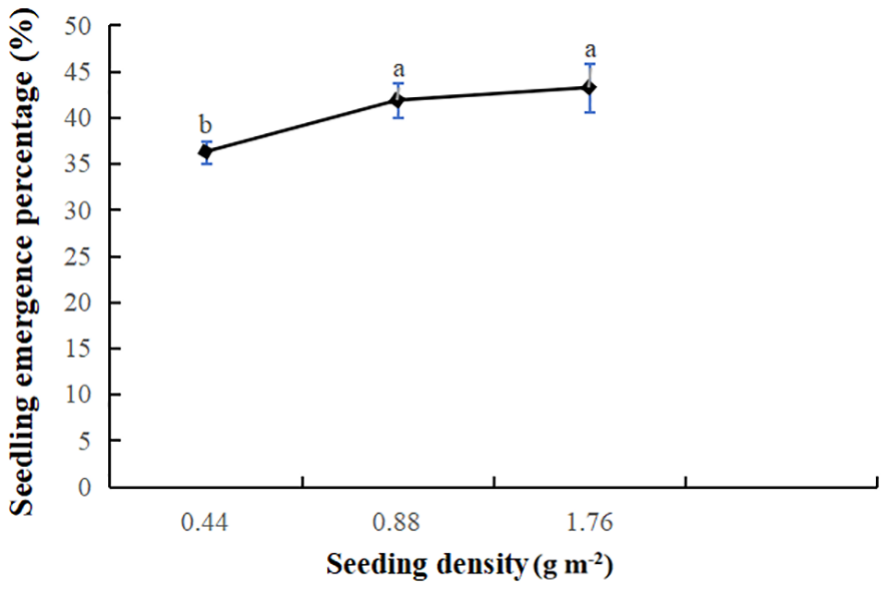
Effects of sowing density on seedling emergence percentage of *V. amurensis* (%). Significant differences at *p* < 0.05 are indicated by different letters.

Adjusting sowing density is crucial for optimizing the expression of productivity. The percentage of seedling emergence at varying sowing densities was illustrated in **Figure 4**. With the increase of sowing density, the seed germination reached the maximum at 1.76 *g* m^-2^. Given the minute size of *V. amurensis* seeds, with a thousand-grain weight of just 0.44 *g*, the study observed a synergistic interaction between seeding rate and sowing density. However, the sowing density was determined as 0.88 *g* m^-2^ in consideration of seedlings’ subsequent growth.

### 3.3 Effects of plastic film mulching and shading on seed germination of *V. amurensis*

**Figure 5:**
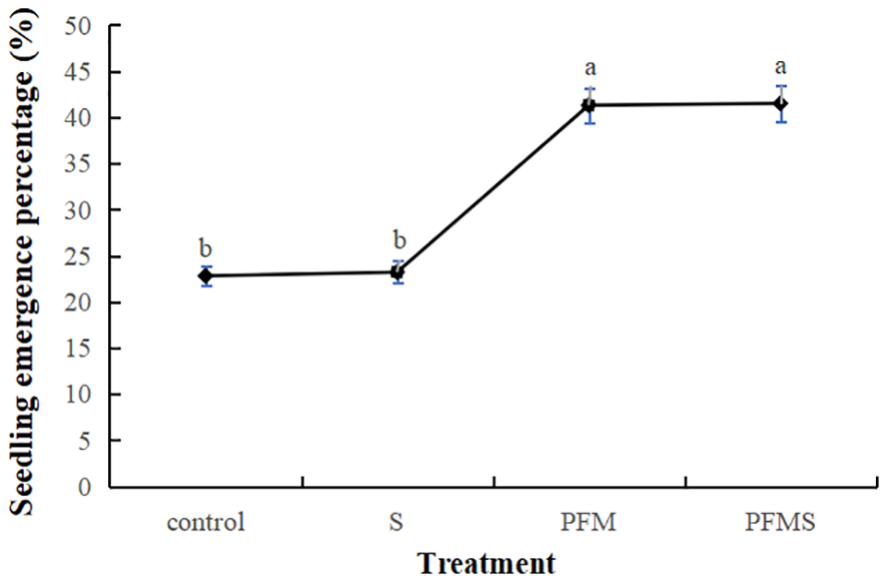
Effects of plastic film mulching and shading on seed germination of *V. amurensis* (*%*). Significant differences at *p* < 0.05 are indicated by different letters.

Data of seed germination percentage influenced by plastic film mulching and shading was represented in **Figure 5**. To maintain moisture in the seedbeds, a certain amount of water was given to the S and control treatments at irregular time. The seedling emergence percentages were 22.8% for the control group and 23.2% for the group with shading alone, indicating that shading didn’t significantly affect seed germination. In contrast, plastic film mulching displayed important effect on seed germination, seedling emergence percentage were 41.5% and 41.3% for the treatments of plastic film mulching combined with shading and plastic film mulching.

### 3.4 Effects of transplanting time on plant growth of *V. amurensis*

The initial transplanting of *V. amurensis* was conducted in early July coinciding with the emergence of the fourth euphylla leaf. Subsequent transplanting occurred in late September 2019 (terminal stage of plant), and in early April 2020 (germination stage of plant). Survival rate was 73% in early July and lower than those (both 98%) of other transplanting time. However, based on minimal damage and good growth vigor, the transplanting time of late September was deemed optimal.

### 3.5 Effects of topping on plant growth of *V. amurensis* in different growth periods

**Figure 6:**
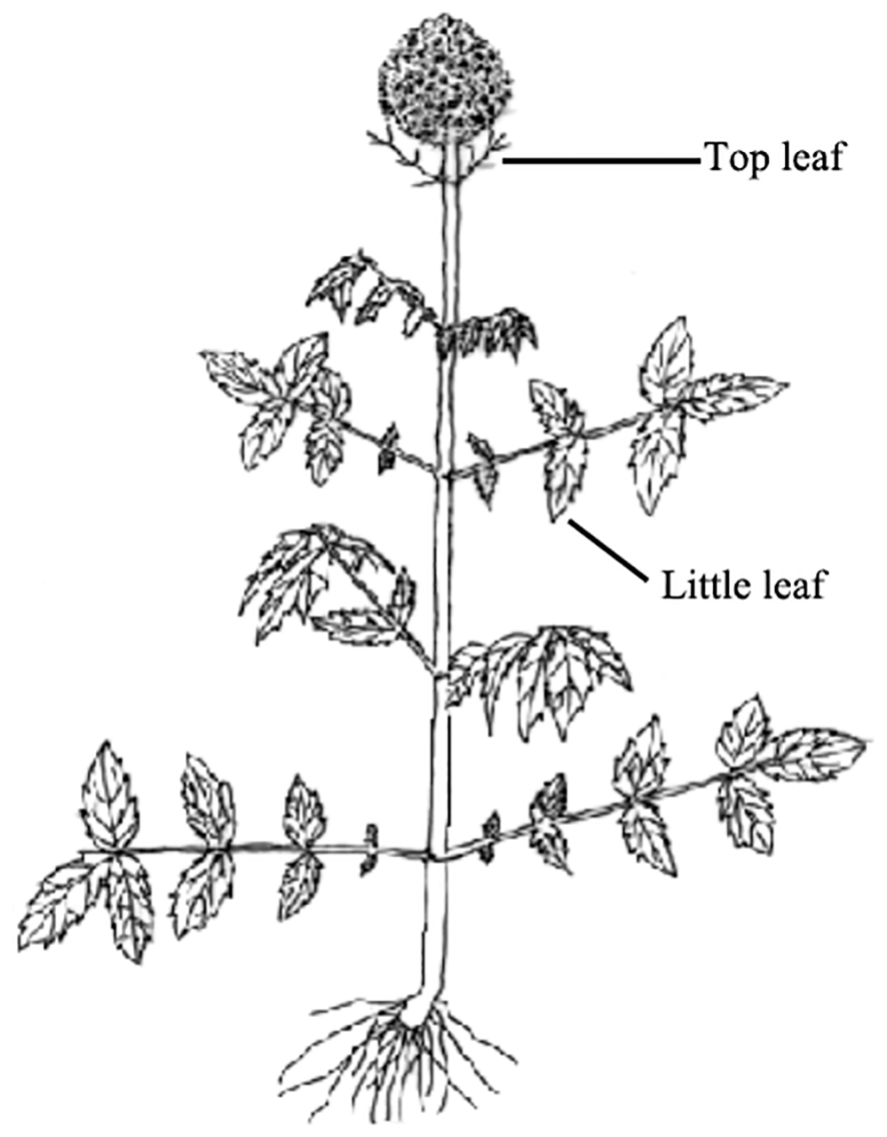
A schematic representation illustrates the morphological characteristics of the original *V. amurensis* plant.

Topping of *V. amurensis* was implemented during the stooling period. Morphological parameters, including the length and width of the top leaf (LTL and WTL), little leaf (LLL and WLL), length of petiole (LP), number of radical leaves (NRL), number of roots (NR), root length (RL), root diameter (RD), stem diameter (SD), and number of buds (NB), were measured during the fruit ripening, late fruit, and withering period (**Table 1**, **Figure 6**). Compared with the control group, the number of radical leaves increased significantly from 11 to 13.67 during the fruit ripening period, and 18.07 to 21.87 during the late fruit period. Additionally, the number of buds and roots in the withering period increased by 1.1 and 25.2 respectively, compared to the control (**Table 1**).

**Table 1:** Effects of topping on indexes of plant growth in different periods (*x̅* ± *s*, *n*=30).

Group	Length of top leaf (LTL, cm)	Width of top leaf (WTL, cm)	Length of little leaf (LLL, cm)	Width of little leaf (WLL, cm)	Length of petiole (LP, cm)	Number of radical leaves (NRL)	Stem diameter (SD, mm)	Number of bud (NB)	Root length (RL, cm)	Root diameter (RD,mm)	Number of root (NR)
Control (fruit ripening period)	7.20	4.30	4.93	3.11	14.12	11.00	9.37	–	–	–	–
Topping (fruit ripening period)	7.22	4.32	4.95	3.12	14.43	13.67	9.46	–	–	–	–
Variability	ns	ns	ns	ns	ns	**	ns				
Control (late fruit period)	4.50	3.17	3.73	2.37	11.69	18.07	–	–	–	–	–
Topping (late fruit period)	4.46	3.18	3.70	2.36	11.97	21.87	–	–	–	–	–
Variability	ns	ns	ns	ns	ns	**					
Control (withering period)	–	–	–	–	–	–	–	5.40	34.80	2.26	209.60
Topping (withering period)	–	–	–	–	–	–	–	6.50	36.02	2.29	234.81
Variability								**	ns	ns	**

^**^ means 0.01 < p ≤ 0.05; ns means no significant difference. Significance level was set as p < 0.05.

### 3.6 Effects of fertilizers on plant growth of *V. amurensis* in withering period

Plants treated with varying levels of N, P, and K were harvested during the withering period, the data for number of roots and buds, root length and root diameter are presented in **Table 2**. For length of roots, generally, fertilization provided the significant improvements. Compared with the control, the average length of roots in the N, P, and K fertilizer groups increased by 1.98 cm, 1.26 cm and 0.51 cm, respectively. Besides, compared with the control group, the root diameter increased by 0.10 mm, 0.13 mm and 0.06 mm, respectively, in the N, P, and K fertilized groups.

**Table 2:** Effects of different fertilization treatment on indexes of plant growth in withering period (*x̅* ± *s*, n=10).

Group	Number of roots (NR)	Root length (RL, cm)	Root diameter (RD, mm)	Number of buds (NB)
Control	209.63	32.61^d^	2.23^b^	5.27
N	232.06	34.59^ab^	2.33^a^	5.47
P	217.02	33.12^cd^	2.36^a^	5.93
K	217.57	33.87^c^	2.29^ab^	5.53
Variability	ns	**	**	ns
LSD	–	± 0.05	± 0.05	–

LSD is the least significant difference between the values in the same column. ^**^ means 0.01 < p ≤ 0.05; ns means no significant difference. Significant differences among different fertilization treatment are indicated by different letters. Significance level was set as p < 0.05.

### 3.7 Effects of topping on biomass and active constituents of *V. amurensis*

In comparison to the control group, the contents of valtrate, valepotriates and essential oil in the topping group were 0.39 mg g^-1^, 28.98 mg g^-1^, and 13.21 mg g^-1^, respectively. Topping had no significant effect (*P* > 0.05) on any of the active constituents contents in this study (**Table 3**). However, the yields of valtrate, valepotriates and essential oil in the topping group were substantially higher, at 10.91, 809.51 and 395.64 g per plant, respectively. Topping played an important role on the significant increase for yields of active constituents compared to the control group (*P* < 0.05 or *P* < 0.01). Additionally, topping significantly influenced both the fresh and dry biomass of the roots and rhizomes (*P* < 0.01), yet it did not exert any significant effect on the drying rate in our study.

**Table 3:** Effects of topping on biomass of root and rhizome, active constituents of *V. amurensis* (*x̅* ± *s*, *n*=10).

Group	Valtrate		Valepotriates		Essential oil		Fresh biomass (mg per plant)	Dry biomass (mg per plant)	Drying rate (%)
	(mg g^-1^)	(mg per plant)	(mg g^-1^)	(mg per plant)	(mg g^-1^)	(mg per plant)			
Control	0.37	8.81	27.53	647.26	13.49	314.16	90.26	23.53	26.07
Topping	0.39	10.91	28.98	809.51	13.21	395.64	107.31	27.92	26.03
Variability	ns	**	ns	***	ns	***	***	***	ns

^***^ means p ≤ 0.01; ^**^ means 0.01 < p ≤ 0.05; ns means no significant difference. Significance level was set as p < 0.05.

### 3.8 Effects of nitrogen, phosphorus and potassium fertilizers on biomass and active constituents of *V. amurensis*

**Figure 7:**
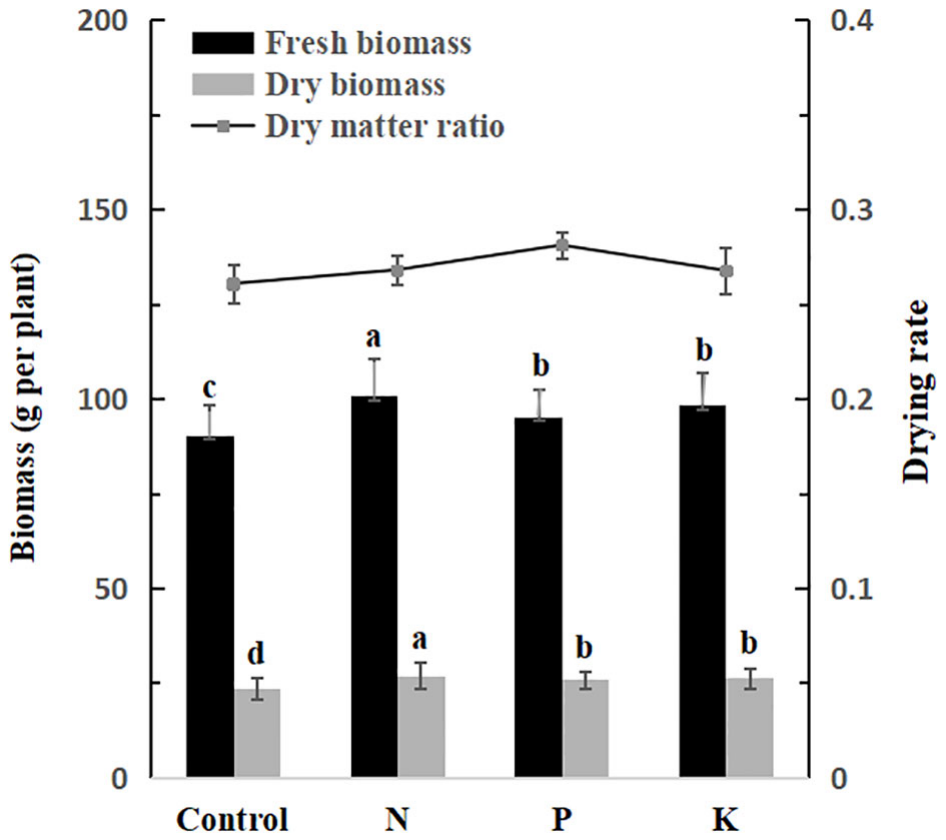
Root fresh biomass (black columns), dry biomass (grey columns) and root drying rate (connected scatter plots) under different fertilizers in in withering period. Significant differences at *p* < 0.05 are indicated by different letters.

Nitrogen, P and K fertilization affected both the fresh and dry biomass of the roots and rhizomes, however, the drying rate of roots and rhizomes didn’t show any significant effects in this study (**Figure 7**). Data revealed the N fertilizer improved the biomass significantly compared with other groups. Both P and K fertilization also increased the biomass of the roots and rhizomes, with no significant difference observed between the two. The average fresh and dry root biomass values for the control, N, P and K treatments were 90.26 and 23.53 g per plant, 100.73 and 27.00 g per plant, 95.28 and 25.83 g per plant and 98.39 and 26.33 g per plant, respectively. No significant difference was found among the drying rates associated with the various fertilizers.

The content and yield of active constituents of *V. amurensis* were evaluated in relation to N, P and K fertilization treatments (**Table 4**). Nitrogen fertilizer notably elevated the content of the valtrate (0.39 mg g^-1^) and valepotriates (28.61 mg g^-1^), marking the highest levels among the treatments. In contrast, P and K fertilizers almost showed no significant difference on contents of active constituents compared to the control. Nevertheless, all three types of fertilization significantly increased the yields of valtrate and valepotriates to varying extents. For valtrate and valepotriates, the yields were 10.46 and 772.32 mg per plant, 9.94 and 732.24 mg per plant, 9.14 and 683.21 mg per plant respectively, for N, P and K treatments. Furthermore, the average yields of valtrate and valepotriates were higher for N fertilizer than K fertilizer group, and were lowest in the P fertilizer group. However, the three fertilizers had no significant effects on the content and yield of essential oil in our study.

**Table 4:** Effects of nitrogen, phosphorus and potassium fertilizers on active constituents of *V. amurensis* (*x̅* ± *s*, *n*=10).

Group	Valtrate		Valepotriates		Essential oil	
	(mg g^-1^)	(mg per plant)	(mg g^-1^)	(mg per plant)	(mg g^-1^)	(mg per plant)
Control	0.37^b^	8.81^d^	27.53^b^	647.26^d^	13.49^a^	314.16^a^
N	0.39^a^	10.46^a^	28.61^a^	772.32^a^	10.94^c^	294.66^b^
P	0.35^c^	9.14^c^	26.45^c^	683.21^c^	11.13^c^	285.71^b^
K	0.38^b^	9.94^b^	27.81^b^	732.24^b^	12.27^b^	320.35^a^
Variability	**	**	**	**	**	**
LSD	± 0.05	± 0.08	± 0.05	± 0.10	± 0.08	± 0.06

LSD is the least significant difference between the values in the same column. ** means 0.01 < p ≤ 0.05; ns means no significant difference. Significant differences among different fertilization treatment are indicated by different letters. The significance level was set as p < 0.05.

## 4 Discussion

### 4.1 Seed germination of *V. amurensis*

In this study the effects of various agricultural practices on the seed germination of *V. amurensis* were evaluated. Sowing time was identified as a critical factor affecting germination, with lower temperatures potentially delaying germination and reducing the germination percentage (Liu et al., 2023). The thermal conditions at different sowing times were found to significantly influence the levels of total soluble proteins, Fe and tannin (Sondeep et al., 2012). In addition, the seeds of *V. amurensis* possess dormant characteristics, so sowing should be conducted at the appropriate temperature without delay (Porceddu et al., 2017; Carol and Jerry, 2020). Herein, late April and early May have been determined as the optimal sowing time for *V. amurensis* in Heilongjiang Province.

The germination percentage constantly increased with higher sowing density, which is a common observation for many species as sowing density typically does not affect establishment likelihood (Mark and Richard, 2010). A synergistic effect of high density of seedling with germination might play a role because the seed of *V. amurensis* was so tiny and the seedling was tender. Although more seedlings will be obtained in the case of higher seeding density, too many seedlings are prone to wilting in high-temperature, high-humidity environments increase susceptibility to diseases and pests (Luo, 2019). Considering of the seedling’s transplant, the sowing density was determined as 0.88 g m^-2^.

Polyethylene mulch is known as an efficient way to strongly boost agricultural output in the past few years (Zhang et al., 2018; Yan et al., 2024). The percentage of seedling emergence and the survival rate of seedling of *V. amurensis* are low in cold and arid environment due to the plant’s dormancy characteristics. Mulches own so many beneficial effects on soil moisture, soil and air temperature, soil water availability, and root growth (Thakur et al., 2019). In our study, the percentage of seedling emergence with the treatment of mulches was significantly higher than that without mulches. Meanwhile, our results show that germination time of all seeds using mulches was 5 days earlier than control group, entire germination time was also 10 days shorter than that lack of mulches.

### 4.2 Plant growth, yield and quality influenced by topping

Topping is recognized as an important cultivating measure for improving the agricultural product quality (Qin et al., 2020). Removing the top bud can stimulate the growth and development of leaves, increase the leaf dimensions and area, slow down the plant height and stem girth, and foster root development (Qiu, 2013; Kaori et al., 2021). According to our study, branch topping does not affect the active constituents contents of *V. amurensis*, at the same time, the drying rate maintains constant in plant growth periods. However, topping plays an important role on the significant increase for yields of active constituents, but also lead to an increase of the fresh and dry biomass of the roots and rhizomes due to removal of new branch sites on the plant. Similarly, topping also plays an important role in the growth period and the accumulation of active components of many medicinal plants, such as *Polygonatum cyrtonema*, *Nicotiana tabacum* L., etc (Fu et al., 2013; Tang et al., 2023). Herein, topping encourages the sprouting of new buds and leaf growth, with the most pronounced effects observed in the fresh and dry biomass, attributed to enhanced photosynthesis resulting from altered plant growth dynamics.

### 4.3 Plant growth, yield and quality influenced by nitrogen, phosphorus and potassium fertilizers

In our study, the application of N, P and K fertilizers enhanced plant growth and increased the fresh and dry root biomass of *V. amurensis*. However, the drying rate of roots and rhizomes didn’t show any significant effect compared with the control. The drying rate of underground parts of different plants can vary significantly across different years and periods, which may be related to the growth of plants and soil moisture content in this period (Gong, 2021). Notably, N fertilizer showed the most significant effect on root length, and the fresh and dry biomass of *V. amurensis*, with significant differences with respect to P and K fertilizers. This effect is attributed to nitrogen’s role in accelerating cell division and differentiation, thereby promoting overall plant growth. Nitrogen, P and K fertilizer play important roles in growth and development of most plants, however, different effects are also revealed in growth periods using fertilizers (Jiang et al., 2018a). Therefore, these differences among fertilizer types may due to the different mode of action, and N application appears the most appropriate for improving both the growth index and biomass for *V. amurensis*.

As a traditional Chinese medicinal plant, *V. amurensis* contains numerous active chemical compounds, and two types of active compounds were measured in this study. Nitrogen fertilizer played the significant influence on the content of valepotriates and valtrate, however, P and K fertilizers did not increase the content of active compounds with the growth of the plant. Nitrogen supply levels had different effects on the accumulation of effective components in different medicinal plants. High nitrogen level can significantly increase the accumulation of total saponins and swertiamarin, while low nitrogen levels can promote the accumulation of saponins in *Gynostemma pentaphyllum* (Thunb.) Makino (Luo and Fu, 2020). Meanwhile, three types of fertilizers all increased the yields of valepotriates and valtrate. The different trends between the content and yields of valepotriates implies that growth rate of plant may exceed the accumulation of active compounds, with primary metabolites decreasing and secondary metabolites accumulating from the growth to the withering stage (Tan et al., 2023).

However, the application of N, P and K fertilizers had no significant influence on the content of essential oil in *V. amurensis*. Similar findings have been reported for the active compounds in *Rheum tanguticum* when treated with nitrogen fertilizer (Xiong et al., 2019). For most plant species, the nutrient threshold for secondary metabolic biosynthesis may different, and the impact factors may also variable.

## 5 Conclusion

In our study, sowing time is determined between the end of April and early May, and the sowing density was set as 0.88g m^-2^. Flat soil surface with plastic film mulching system is the better cultivation practice for *V. amurensis*. The transplanting time was made sure owing to slight damage and good growth vigor in withering period. Topping significantly increased the some growth indexes (the number of radical leaves, buds and roots in withering period) and biomass of *V. amurensis* but had no significant effects on the drying rate. Moreover, fertilizers also play important effects on the plant growth, biomass and active constituents of *V. amurensis*. The N fertilizer application was identified as the most appropriate in consider of the fresh and dry biomass of the roots and rhizomes. Meanwhile, the yields of valtrate and valepotriates were increased to maximum by applying N fertilizer. It is crucial to explore important effects of soil moisture, soil and air temperature on seed germination. Due to limitations in experimental site, this study did not conduct before NPK fertilization influence and active components analysis. Future research will delve into these aspects for a more in-depth understanding of seed germination characteristics of *V. amurensis*.

## Data availability statement

The raw data supporting the conclusions of this article will be made available by the authors, without undue reservation.

## Ethics statement

The author states that *Valeriana amurensis* involved in this study do not involve ethical relations. Experimental research on plants, including the collection of plant material, complies with relevant institutional, national, and international guidelines and legislation.

## Author contributions

JW: Data curation, Formal analysis, Investigation, Methodology, Writing – original draft. DL: Data curation, Formal analysis, Resources, Software, Writing – original draft. JH: Formal analysis, Methodology, Supervision, Validation, Writing – review & editing. RZ: Data curation, Formal analysis, Resources, Visualization, Writing – original draft. XD: Conceptualization, Funding acquisition, Project administration, Writing – review & editing.
